# Supplementary material for: Neighborhood environment, social cohesion, and epigenetic aging
Source: Aging (Albany NY). 2021 Mar 14;13(6):7883–99. doi: 10.18632/aging.202814 (PMC8034890; doi:10.18632/aging.202814)
Supplement: Supplementary Tables [file aging-13-202814-s002.pdf]

SUPPLEMENTARY TABLES

Supplementary Table 1. Definitions and distributions (mean and standard deviation (SD)) for the 19 neighborhood quality indicators assessed by two trained assessors for a select number of census block groups in the Detroit Neighborhood Health Study.

Neighborhood quality indicator	Metric as evaluated by trained assessor	Mean	SD
HQ1	Are there any buildings with broken windows, boarded up windows, or boarded up doors? Percent of sampled block group segments within Neighborhood that have "Yes" for this question.	34.2	14.1
HQ2	Are there any buildings with outside damage that can only be corrected by major repairs such as siding, shingles, boards, brick, concrete, and stucco? Percent of sampled block group segments within Neighborhood that have "Yes" for this question.	29.7	14
HQ3	Are there any entirely vacant buildings? Percent of sampled block group segments within Neighborhood that have "Yes" for this question.	34	11.8
HQ4	Are there any empty, vacant lots? Percent of sampled block group segments within Neighborhood that have "Yes" for this question.	32.6	21.4
HQ5	Are there any construction sites? Percent of sampled block group segments within Neighborhood that have "Yes" for this question.	2.4	2.5
HQ6	Is there a community garden? Percent of sampled block group segments within Neighborhood that have "Yes" for this question.	0.566	0.873
HQ7	Is there graffiti (non-art)? Percent of sampled block group segments within Neighborhood that have "Yes" for this question.	17.2	10.3
HQ8	Are the street and sidewalk clean? Percent of sampled block group segments within Neighborhood that have "Yes" for this question.	73.3	14.1
HQ9	Are there any big, mature trees? Percent of sampled block group segments within Neighborhood that have "Yes" for this question.	83.1	9.64
HQ10	Is there heavy traffic volume? Percent of sampled block group segments within Neighborhood that have "Yes" for this question.	30.2	10.8
HQ11	Is the street in poor condition? Percent of sampled block group segments within Neighborhood that have "Yes" for this question.	33	11.4
HQ12	Is the sidewalk in poor condition? Percent of sampled block group segments within Neighborhood that have "Yes" for this question.	58.7	15.2
HQ13	Is the street noisy? Percent of sampled block group segments within Neighborhood that have "Yes" for this question.	26.9	10.3
HQ14	Are there people visible on the street? Percent of sampled block group segments within Neighborhood that have "Yes" for this question.	13.7	8.68
HQ15	Are there any abandoned cars? Percent of sampled block group segments within Neighborhood that have "Yes" for this question.	6.57	4.04
HQ16	Are any of the following signs visible? A. Neighborhood or Crime Watch. B. Security warning signs. Percent of sampled block group segments within Neighborhood that have "Yes" for this question.	51.8	14.4
HQ17	Are there any tobacco product advertising signs visible? Percent of sampled block group segments within Neighborhood that have "Yes" for this question.	1.65	1.88
HQ18	Are there any alcohol advertising signs visible? Percent of sampled block group segments within Neighborhood that have "Yes" for this question.	2.49	2.01
HQ19	Are there any For Sale OR For Lease OR For Rent signs visible? Percent of sampled block group segments within Neighborhood that have "Yes" for this question.	18.6	6.72

Supplementary Table 2. Loadings for each of the top eight principal components.

	PC1	PC2	PC3	PC4	PC5	PC6	PC7	PC8
HQ1	-0.35	0.10	-0.08	0.06	-0.12	0.07	-0.02	0.00
HQ2	-0.36	0.10	0.00	-0.03	-0.11	0.05	0.04	0.21
HQ3	-0.29	0.19	-0.18	0.08	-0.15	0.20	0.00	-0.14
HQ4	-0.35	-0.04	0.08	-0.14	0.17	0.07	-0.26	0.15
HQ5	-0.02	-0.16	-0.39	0.16	0.43	0.48	0.10	-0.08
HQ6	-0.08	-0.01	0.14	0.73	0.18	-0.25	0.12	-0.30
HQ7	-0.23	-0.21	0.19	0.34	-0.23	0.15	0.08	0.35
HQ8	0.33	0.06	0.01	0.21	-0.18	-0.06	0.22	-0.04
HQ9	0.12	0.28	0.02	0.16	0.57	-0.25	-0.16	0.59
HQ10	0.01	-0.45	0.11	-0.10	0.27	0.07	0.11	-0.02
HQ11	-0.30	0.07	0.11	0.20	0.16	-0.16	-0.06	-0.28
HQ12	-0.32	0.20	0.03	-0.06	0.15	0.08	0.02	-0.05
HQ13	0.03	-0.47	0.02	-0.02	0.20	0.14	-0.11	-0.20
HQ14	-0.06	-0.42	-0.03	0.10	-0.16	-0.12	0.34	0.43
HQ15	-0.22	0.12	-0.21	-0.23	0.19	-0.20	0.75	-0.03
HQ16	0.28	0.24	-0.22	0.01	0.03	0.05	0.14	-0.03
HQ17	-0.05	-0.17	-0.52	-0.07	0.08	-0.30	-0.20	-0.02
HQ18	-0.13	-0.20	-0.40	0.05	-0.20	-0.50	-0.22	-0.05
HQ19	0.08	0.09	-0.45	0.33	-0.16	0.35	-0.08	0.20
Eigenvalue	6.6	3.7	2.5	1.3	1.0	0.8	0.6	0.6
% of variation explained	35.0%	19.3%	12.7%	7.0%	5.0%	4.3%	3.0%	2.8%

Supplementary Table 3. Association between neighborhod characteristics aand epigenetic aging.

	All		Women		Men	
	Model A[A] β (95% CI)	Model B[B] β (95% CI)	Model A β (95% CI)	Model B β (95% CI)	Model A β (95% CI)	Model B β (95% CI)
Neighborhood poverty						
Horvath age acceleration	0.7 (-0.3, 1.8)	0.8 (-0.2, 1.8)	0.7 (-0.7, 2.1)	0.7 (-0.7, 2.0)	0.1 (-1.5, 1.7)	0.2 (-1.4, 1.9)
Hannaum age acceleration	0.7 (-0.3, 1.7)	0.8 (-0.1, 1.7)	0.9 (-0.5, 2.3)	0.6 (-0.6, 1.9)	-0.3 (-1.7, 1.2)	0.3 (-1.2, 1.8)
PhenoAge acceleration	1.0 (-0.3, 2.4)	1.1 (-0.2, 2.3)	1.4 (-0.4, 3.3)	1.0 (-0.8, 2.7)	-0.3 (-2.2, 1.5)	0.6 (-1.3, 2.5)
Neighborhood social cohesion						
Horvath age acceleration	0.0 (-0.5, 0.5)	0.0 (-0.4, 0.5)	0.3 (-0.4, 0.9)	0.1 (-0.4, 0.7)	-0.3 (-1.0, 0.5)	-0.1 (-0.9, 0.6)
Hannaum age acceleration	-0.1 (-0.6, 0.4)	-0.1 (-0.5, 0.3)	0.2 (-0.5, 0.8)	0.1 (-0.5, 0.6)	-0.6 (-1.2, 0.1)	-0.5 (-1.2, 0.1)
PhenoAge acceleration	-0.3 (0.9, 0.3)	-0.2 (-0.8, 0.4)	0.1 (-0.7, 1.0)	0.2 (-0.6, 0.9)	-0.7 (-1.6, 0.1)	-0.7 (-1.5, 0.1)

[A]Models are adjusted for race/ethnicity, education level, employment, smoking status, alcohol intake, years residing in current neighborhood.

[B]Models are adjusted for race/ethnicity, education level, employment, smoking status, alcohol intake, years residing in current neighborhood, and cell proportion estimates.

Supplementary Table 4. Association between principal components and epigenetic aging.

	All		Women		Men	
	Model A[A] β (95% CI)	Model B[B] β (95% CI)	Model A β (95% CI)	Model B β (95% CI)	Model A β (95% CI)	Model B β (95% CI)
			Horvath age acceleration			
PC1	-0.1 (-0.6, 0.3)	-0.3 (-0.6, 0.1)	-0.1 (-0.6, 0.4)	-0.3 (-0.8, 0.2)	0.2 (-0.5, 0.9)	0.1 (-0.6, 0.7)
PC2	0.0 (-0.5, 0.6)	0.1 (-0.3, 0.6)	-0.1 (-0.8, 0.6)	-0.3 (-0.9, 0.4)	0.1 (-0.7, 0.9)	0.5 (-0.3, 1.2)
PC3	0.7 (-0.1, 1.3)	0.4 (-0.3, 1.0)	0.7 (-0.2, 1.6)	0.5 (-0.3, 1.4)	-0.1 (-1.1, 1.0)	-0.4 (-1.4, 0.6)
PC4	-0.3 (-1.2, 0.7)	-0.1 (-0.9, 0.8)	0.0 (-1.3, 1.3)	0.1 (-1.1, 1.3)	-0.4 (-1.8, 1.0)	-0.4 (-1.7, 1.0)
PC5	0.0 (-1.1, 1.1)	-0.4 (-1.4, 0.6)	0.2 (-1.2, 1.6)	-0.4 (-1.7, 0.9)	0.2 (-1.2, 1.6)	-0.4 (-2.1, 1.4)
PC6	1.0 (-0.2, 2.1)	0.9 (-0.1, 2.0)	**1.6 (0.0, 3.1)**	**1.5 (0.0, 2.9)**	0.0 (-1.7, 1.8)	0.1 (-1.7, 1.9)
PC7	**1.8 (0.4, 3.1)**	0.8 (-0.5, 2.1)	0.9 (-0.9, 2.8)	-0.4 (-2.3, 1.6)	1.9 (-0.1, 4.0)	1.6 (-0.4, 3.6)
PC8	0.1 (-1.4, 1.7)	-0.1 (-1.5, 1.3)	-0.1 (-2.1, 1.8)	0.0 (-1.8, 1.9)	-0.6 (-3.0, 1.9)	-0.5 (-3.0, 2.0)
			Hannum age acceleration			
PC1	-0.2 (-0.6, 0.2)	-0.2 (-0.6, 0.1)	-0.2 (-0.7, 0.3)	-0.3 (-0.8, 0.1)	0.3 (-0.3, 0.8)	0.2 (-0.4, 0.7)
PC2	0.1 (-0.4, 0.6)	0.1 (-0.3, 0.6)	-0.1 (-0.8, 0.6)	-0.3 (-0.9, 0.4)	0.3 (-0.5, 1.0)	0.4 (-0.3, 1.1)
PC3	0.3 (-0.3, 1.0)	-0.1 (-0.6, 0.6)	0.2 (-0.7, 1.1)	-0.0 (-0.8, 0.7)	-0.0 (-1.0, 0.9)	-0.5 (-1.4, 0.5)
PC4	-0.1 (-1.0, 0.7)	0.1 (-0.6, 0.9)	0.0 (-1.2, 1.3)	0.3 (-0.8, 1.4)	-0.1 (-1.4, 1.2)	-0.1 (-1.3, 1.1)
PC5	-0.0 (-1.1, 1.0)	-0.3 (-1.2, 0.6)	0.1 (-1.2, 1.5)	-0.5 (-1.7, 0.7)	0.8 (-0.8, 2.4)	0.6 (-0.9, 2.2)
PC6	0.4 (-0.8, 1.5)	0.4 (-0.7, 1.4)	1.1 (-0.5, 2.6)	0.6 (-0.8, 2.0)	-0.7 (-2.3, 0.9)	0.1 (-1.6, 1.7)
PC7	**1.7 (0.4, 3.0)**	0.6 (-0.6, 1.8)	1.5 (-0.3, 3.3)	-0.2 (-2.0, 1.6)	1.1 (-0.8, 2.9)	1.5 (-0.3, 3.3)
PC8	0.3 (-1.2, 1.8)	-0.1 (-1.5, 1.2)	0.9 (-1.0, 2.8)	0.9 (-0.8, 2.6)	-1.9 (-4.1, 0.2)	-2.3 (-4.4, -0.1)
			PhenoAge acceleration			
PC1	-0.3 (-0.8, 0.2)	-0.4 (-0.8, 0.1)	-0.3 (-1.0, 0.4)	-0.5 (-1.1, 0.2)	0.3 (-0.5, 1.0)	0.2 (-0.6, 0.9)
PC2	-0.1 (-0.8, 0.6)	-0.1 (-0.7, 0.5)	-0.4 (-1.3, 0.5)	-0.6 (-1.5, 0.2)	0.1 (-0.8, 1.0)	0.2 (-0.7, 1.1)
PC3	0.4 (-0.4, 1.3)	0.0 (-0.8, 0.8)	0.3 (-0.9, 1.5)	-0.1 (-1.2, 1.0)	-0.1 (-1.8, 0.5)	-0.6 (-1.7, 0.6)
PC4	-0.6 (-1.8, 0.5)	-0.4 (-1.4, 0.7)	-0.2 (-1.8, 1.5)	-0.1 (-1.6, 1.5)	-0.9 (-2.5, 0.7)	-1.1 (-2.6, 0.4)
PC5	-0.6 (-1.9, 0.7)	-0.8 (-2.0, 0.5)	-0.6 (-2.4, 1.2)	-1.0 (-2.7, 0.6)	-0.2 (-2.3, 1.9)	-0.5 (-2.5, 1.5)
PC6	0.4 (-1.1, 1.9)	0.5 (-0.9, 1.9)	1.5 (-0.6, 3.6)	0.9 (-1.0, 2.8)	-1.0 (-3.0, 1.0)	-0.3 (-2.3, 1.8)
PC7	**2.1 (0.4, 3.8)**	0.8 (-0.8, 2.5)	2.4 (-0.0, 4.9)	0.3 (-2.3, 2.7)	0.2 (-2.2, 2.6)	0.4 (-2.0, 2.7)
PC8	-0.0 (-1.9, 1.9)	-0.7 (-2.5, 1.1)	-0.2 (-2.8, 2.4)	-0.6 (-3.0, 1.8)	-0.8 (-3.7, 2.0)	-0.4 (-3.3, 2.4)

[A]Models are adjusted for race/ethnicity, education level, employment, smoking status, alcohol intake, and years residing in current neighborhood.

[B]Models are adjusted for race/ethnicity, education level, employment, smoking status, alcohol intake, years residing in current neighborhood, and cell proportion estimates.

Supplementary Table 5. Association between neighborhood characteristics and epigenetic aging by neighborhood social cohesion.

	All		Higher social cohesion		Lower social cohesion	
	**Model A[A]**	**Model B[B]**	**Model A**	**Model B**	**Model A**	**Model B**
	**β (95% CI)**	**β (95% CI)**	**β (95% CI)**	**β (95% CI)**	**β (95% CI)**	**β (95% CI)**
Neighborhood poverty						
Horvath age acceleration	0.7 (-0.3, 1.8)	0.8 (-0.2, 1.8)	0.3 (-1.5, 2.1)	0.5 (-1.1, 2.2)	1.3 (-0.0, 2.8)	1.2 (-0.3, 2.7)
Hannaum age acceleration	0.7 (-0.3, 1.7)	0.8 (-0.1, 1.7)	0.3 (-1.2, 1.9)	0.7 (-0.8, 2.2)	1.1 (-0.5, 2.6)	0.7 (-0.8, 2.2)
PhenoAge acceleration	1.0 (-0.3, 2.4)	1.1 (-0.2, 2.3)	0.5 (-1.6, 2.5)	1.2 (-0.9, 3.3)	1.6 (-0.4, 3.7)	0.9 (-1.1, 2.9)
Neighborhood PC7						
Horvath age acceleration	**1.8 (0.4, 3.1)**	0.8 (-0.5, 2.1)	1.3 (-1.4, 3.9)	0.3 (-2.1, 2.7)	**2.1 (0.6, 3.6)**	1.5 (-0.1, 3.1)
Hannaum age acceleration	**1.7 (0.4, 3.0)**	0.6 (-0.6, 1.8)	0.9 (-1.4, 3.2)	-0.0 (-2.2, 2.1)	**2.2 (0.6, 3.8)**	1.3 (-0.3, 3.0)
PhenoAge acceleration	**2.1 (0.4, 3.8)**	0.8 (-0.8, 2.5)	0.8 (-2.2, 3.9)	0.1 (-3.0, 3.2)	**2.3 (0.3, 4.7)**	1.3 (-0.8, 3.5)

[A]Models are adjusted for race/ethnicity, education level, employment, smoking status, alcohol intake, years residing in current neighborhood.

[B]Models are adjusted for race/ethnicity, education level, employment, smoking status, alcohol intake, years residing in current neighborhood, and cell proportion estimates.
